# Prediction of HIV incidence in Xinjiang Uygur autonomous region, China: a time series study

**DOI:** 10.3389/fpubh.2026.1920865

**Published:** 2026-10-14

**Authors:** Tao Zhang, Daren Zhao

**Affiliations:** 1 Department of Hospital Administration Office, First Affiliated Hospital of Xinjiang Medical University, Urumqi, Xinjiang, China; 2 Faculty of Social Sciences and Humanities, Mahidol University, Salaya, Nakhon Pathom Province, Thailand; 3 Department of Quality Control Management, Sichuan Provincial Orthopedics Hospital, Chengdu, Sichuan, China

**Keywords:** China, exogenous variable, HIV, prophet model, random forest, SARIMAX, time series

## Abstract

**Objective:**

In China, Xinjiang Uygur Autonomous Region has a high incidence of HIV, and the situation regarding its prevention and control remains challenging. This study aimed to compare the predictive performance of three time series models, SARIMAX (Seasonal Autoregressive Integrated Moving Average with Exogenous Regressors), Prophet with exogenous variables, and random forest, using rolling validation, and to provide references for HIV prevention and control in Xinjiang.

**Methods:**

We collected 168 months of HIV incidence data in Xinjiang from 2012 to 2025. SARIMAX, Prophet, and random forest were developed with COVID-19 as an exogenous intervention variable. Prophet was tuned via grid search (optimal changepoint.prior.scale=0.1, seasonality.prior.scale=1); random forest was optimized with mtry=3 and 12-month lags selected by PACF. Rolling validation used RMSE, MAE, MAPE, sMAPE, and MASE with Bootstrap 95% CI. SARIMAX failed to converge in the first four windows; therefore, models were compared on windows 5–9 (2021–2025).

**Results:**

The optimal SARIMAX model was SARIMAX(0, 1, 4)(1, 0, 0)(1) with an exogenous COVID-19 intervention variable; its residuals passed the Ljung-Box test (*Q* = 8.8, *p* = 0.7199). The Prophet model incorporated seasonal and periodic components and included COVID-19 as an exogenous variable; Bayesian MCMC inference gave a posterior mean of −0.1064. The random forest model used 12 lagged values, month, and COVID-19 as features, achieving a permutation importance (%IncMSE) of 4.31% for the COVID-19 variable, ranking 10th among 14 features. SHapley Additive exPlanations (SHAP) analysis further showed that COVID-19 ranked 14th (last) with a mean absolute SHAP value of 0.00135. Within windows 5–9 (2021–2025), random forest had the lowest RMSE (0.2211), MAE (0.1722), and MAPE (85.83%); (sMAPE 41.11%, MASE 0.8745). However, Diebold–Mariano tests with Bonferroni correction showed no significant differences among models (all *p* > 0.0167), with small RMSE differences (0.0047–0.0225).

**Conclusions:**

All three models are applicable. Although random forest had the lowest numerical errors, it was statistically indistinguishable from the other models; therefore, model selection should consider interpretability, data-updating frequency, and resource constraints rather than marginal accuracy differences. These findings support HIV surveillance and resource allocation in Xinjiang.

## Introduction

Human immunodeficiency virus (HIV) is a retrovirus that causes severe immune dysfunction by depleting CD4^+^ T lymphocytes, and without timely treatment, patients eventually die from opportunistic infections and malignancies (2). Despite enormous global efforts in HIV treatment and prevention, HIV remains a critical public health issue worldwide (3). According to the 2024 Global AIDS Report, approximately 39.9 million people were living with HIV globally in 2023, with about 1.5 million new cases and 630,000 deaths from HIV related diseases annually (4, 5). The global HIV/AIDS burden is particularly heavy in low income regions, especially in sub Saharan Africa, where more than 60% of HIV related deaths occur.

According to the Law of the People's Republic of China on the Prevention and Control of Infectious Diseases, notifiable infectious diseases are classified into three categories: A, B, and C. HIV is a Category B disease, requiring medical and disease control institutions to report confirmed HIV cases or AIDS patients through the national online direct reporting system within 24 h and to implement necessary treatment and transmission control measures (6). In China, the reported notification rate and HIV related mortality had been slowly increasing over a long period but began to decline in 2018 (7). Nevertheless, population prevalence continues to rise slowly (7). Sexual contact remains the primary mode of HIV transmission. In particular, the proportion of transmission through sex between men increased from 9.1% in 2009 to 23.3% in 2020 (8). In contrast, the proportion of HIV transmission through injecting drug use has decreased (8). Key populations for HIV include men who have sex with men, female sex workers, college students, and older adults (9, 10). Although the Chinese government has implemented policies and allocated substantial resources to HIV prevention and control, great challenges remain.

Xinjiang Uygur Autonomous Region is the largest region in western China and one of the areas with a high burden of tuberculosis and HIV (11). The situation regarding HIV prevention and control remains challenging. Previous studies have shown that both HIV incidence and mortality rates in Xinjiang from 2004 to 2020 were higher than the national average (12). The prevalence of tuberculosis among people living with HIV in this region also exceeded the national average (1). In terms of prevention and treatment, Xinjiang has made substantial efforts. Since 2006, free antiretroviral therapy (ART) has been provided to people living with HIV/AIDS in Xinjiang. Since 2016, the region has further adopted a comprehensive strategy of treating as many as possible, aiming to expand treatment coverage to all eligible individuals (13).

It is critical to perform forecasting and early warning of infectious diseases for their effective prevention and control (14). Specifically, traditional time series models, including ARIMA (15), exponential smoothing (16), and GM(1, 1) (17), have been widely employed in this domain. Furthermore, machine learning models such as XGBoost (18), and LSTM (19), as well as hybrid models like EMD-BPNN (20), ARIMA-LSTM (21), ARIMA-ERNN (22), SARIMA-XGBoost (23), and SARIMA-BPNN (24), are also frequently utilized for infectious disease prediction. In addition, recent studies have modeled infectious disease dynamics using stochastic and seasonal approaches. Akher et al. (25) applied continuous-time Markov chain and stochastic differential equations to simulate malaria transmission and identified key parameters. Akhi et al. (26) developed a periodic stochastic vector-host model and found that outbreak probability is closely related to the timing of introduction of infected individuals. Recent HIV forecasting studies have explored various modeling approaches. Liu et al. (27) developed a SIDMA compartmental model with HIV detection and applied ARIMA models to predict HIV/AIDS epidemic trends in Fujian Province. Wang et al. (28) analyzed 21 years of surveillance data from Huangshi City and employed exponential smoothing time-series models to forecast HIV-related mortality trends for 2025–2028.

However, several research gaps remain. First, existing time series forecasting studies on HIV incidence in Xinjiang have primarily focused on comparing traditional statistical models with deep learning methods (12), and no study to date has incorporated exogenous variables into prediction models, such as SARIMAX, Prophet, or other machine learning approaches. Second, regarding model validation, although rolling validation has been applied in some infectious disease forecasting studies (29, 30), its use for prospective evaluation in HIV prediction research specific to Xinjiang remains limited. Third, uncertainty quantification has not been addressed in Xinjiang HIV incidence forecasting. Fourth, model interpretability remains unexplored, with no application of SHAP or other model-agnostic tools to identify key transmission drivers. Therefore, further advances in HIV incidence prediction research in Xinjiang are needed to fill these gaps.

To address these gaps, this study uses 168 months of monthly HIV incidence data in Xinjiang from January 2012 to December 2025. We applied three models, SARIMAX, Prophet, and random forest, with COVID-19 as an external covariate and rolling window validation for prospective evaluation. The novelty of this study has five aspects. First, in model construction, this study introduces an external covariate (COVID-19) into HIV incidence prediction in Xinjiang, filling the gap of exogenous variable-based prediction in this region. Second, rolling window validation is adopted for prospective evaluation, replacing the traditional train-test split to better simulate real-world forecasting. Third, in comparison framework, this study goes beyond a simple “which model is best” comparison; instead, we explore how model performance depends on data characteristics and application needs, providing guidance for model selection rather than a universally optimal model. Fourth, we report Bootstrap-derived 95% confidence intervals to quantify prediction uncertainty. Fifth, we employ SHAP analysis to enhance model interpretability.

Within this framework, the study aims to (1) compare the predictive performance of the three models, (2) evaluate their forecasting accuracy using rolling validation based on RMSE, MAE, MAPE, sMAPE, and MASE with the Diebold–Mariano test indicating no statistically significant differences among the three models, and (3) provide valuable references for HIV prevention and control in Xinjiang using the prediction results.

## Materials and methods

### Study setting

The Xinjiang Uygur Autonomous Region is located in the northwestern border of China and the hinterland of the Eurasian continent. It borders China's Tibet, Qinghai, and Gansu provinces, and shares borders with eight countries: Mongolia, Russia, Kazakhstan, Kyrgyzstan, Tajikistan, Afghanistan, Pakistan, and India. The region covers an area of 1.6649 million square kilometers, with a land border extending over 5,700 kilometers. It is the provincial-level administrative region with the largest land area, the most neighboring countries, and the longest land border in China. By the end of 2024, the permanent population of the region was 26.228 million. Xinjiang has 14 prefecture-level divisions, including five autonomous prefectures, four prefecture-level cities, and five prefectures, which together comprise 110 county-level divisions (31).

### Data source

We collected data on HIV incidence in Xinjiang Uygur Autonomous Region, China for 168 months from 2012 to 2025. HIV incidence data from January 2012 to December 2020 were extracted from the China Public Health Science Data Center. For January 2021 to December 2025, HIV case counts were extracted from the official website of the Health Commission of Xinjiang Uygur Autonomous Region, and monthly HIV incidence was calculated using annual population data as denominators. Annual population data were extracted from the Statistical Bulletin of National Economic and Social Development of Xinjiang Uygur Autonomous Region for the years 2021 to 2025.

It should be noted that HIV is a class B notifiable infectious disease in China, and its case definition, diagnostic criteria, and reporting requirements are uniform nationwide. Therefore, the two sources have consistent statistical standards, and both ultimately derive from the notifiable infectious disease direct reporting system of the Xinjiang Uygur Autonomous Region Center for Disease Control and Prevention. As the China Public Health Science Data Center has not yet updated its data to cover 2021–2025, the data for this period were supplemented from the official website of the Health Commission of Xinjiang Uygur Autonomous Region.

### Incorporating exogenous variables

To account for the COVID-19 pandemic as a structural disruption while preserving temporal continuity, a binary exogenous variable was introduced. Following the precedent of relevant studies (32), HIV incidence data for the 2020–2022 period were coded as 1, while data for all other years were coded as 0. This variable was incorporated into the forecasting models to capture extreme fluctuations during the pandemic and allow the models to learn from the structural disruption. Consequently, the complete monthly HIV incidence data from 2012 to 2025 were used for analysis. In our study, it should be noted that this binary coding represents a simplified approximation of the pandemic's impact; fine-grained covariates such as mobility, testing volume, and stringency indices were not included due to data availability constraints.

### Research methods

#### SARIMAX model

The ARIMA model was proposed by Box and Jenkins as a classic time series forecasting method. SARIMA extends the ARIMA model to capture both trend and seasonal fluctuations in time series (33). SARIMAX (seasonal autoregressive integrated moving average with exogenous variables) is an extension of the traditional SARIMA model that incorporates exogenous variables. The basic expression of SARIMAX is as follows (34):

$$\Phi (B^{s})\phi (B){\left(1-B\right)}^{d}{\left(1-B^{s}\right)}^{D}y_{t}=\theta (B)\Theta (B^{s})\varepsilon_{t}+\beta X_{t}$$

where *y*_*t*_ is the HIV incidence rate at time *t, X*_*t*_ is the exogenous variable (COVID-19 intervention variable, coded as 1 for the 2020–2022 period and 0 otherwise), β is the regression coefficient of the exogenous variable, ϕ(*B*) is AR(p), θ(*B*) is MA(q), Φ(*B*^*s*^) is SAR(P), Θ(*B*^*s*^) is SMA(Q), *d* and *D* are the orders of non-seasonal and seasonal differencing, respectively, s is the seasonal period length (*s* = 12), and ε_*t*_is the white noise process.

SARIMAX modeling consists of four main steps (35–37). The first step is testing the stationarity of the original time series. The Kwiatkowski Phillips Schmidt shin (KPSS) test, the Augmented dickey fuller (ADF) test, or a time series plot can be used to determine stationarity. The second step is model identification. The autocorrelation function (ACF), the partial autocorrelation function (PACF), and the number of differencing operations are used to preliminarily estimate the relevant model parameters. The third step is model diagnostics and testing. Model diagnostics employed the Ljung-Box test to assess the white noise property of residuals, along with residual time series plots, ACF plots, Q-Q plots, and histograms to verify the independent and identically distributed assumption. The fourth step is model selection. The optimal model is selected based on the minimum Akaike information criterion (AIC), Bayesian information criterion (BIC), and the Ljung-Box test.

### Prophet model with exogenous variables

The Prophet model is a time series forecasting method developed by Facebook based on additive principles. This model relies on an additive regression framework and is well suited to fit nonlinear trends that incorporate seasonality and holiday effects (38–40).

The Prophet model with an exogenous variable can be expressed as follows:

$$y(t)=g(t)+s(t)+h(t)+\beta X_{t}+\varepsilon_{t}$$

In this formula, *g(t)* is the trend term that captures non periodic changes in the time series. *s(t)* represents periodic variations. *h(t)* accounts for the impact of holidays on the time series. *X*_*t*_ is the exogenous variable (covid), β is the regression coefficient of the exogenous variable, ε_*t*_ is the noise term that reflects fluctuations not captured by the model. In this study, a binary exogenous variable *X*_*t*_ (coded as 1 for 2020–2022 and 0 otherwise) was incorporated into the model as an additional regressor. Parameter estimation is performed using the Stan probabilistic programming language. Model optimization is achieved by adjusting parameters such as trend flexibility and seasonal intensity.

In our study, we performed a grid search over changepoint.prior.scale (0.01, 0.05, 0.10) and seasonality.prior.scale (1, 5, 10) on the validation period 2017–2019, and selected the pair (0.1, 1) that achieved the lowest RMSE.

### Random forest

Random forest (RF) is an ensemble machine learning method proposed by Breiman (41). Its principle involves generating multiple decision trees through Bootstrap sampling and averaging their predictions to reduce variance (42). The main steps of RF modeling are as follows (42): first, the Bootstrap method is used to draw samples with replacement from the original training set while randomly selecting a subset of features. Second, a decision tree is trained based on each Bootstrap sample and its corresponding feature subset. Finally, the predictions from all decision trees are averaged to produce the final forecast. In this study, lagged HIV incidence rates from 1 to 12 months, a binary exogenous variable for COVID-19 (coded as 1 for the 2020–2022 period and 0 otherwise), and month were used as feature variables to construct a random forest regression model for prediction.

In our study, we tuned the mtry parameter via tune RF(), selected lags up to 12 months based on PACF while excluding longer lags (e.g., 24 months) given the 168-month series length, and performed SHAP analysis using the *iml* R package to enhance model interpretability.

### Prediction performance evaluation

In this study, a rolling time window validation method combined with error metrics was used to evaluate model predictive performance. Additionally, the Diebold–Mariano (DM) test was employed to conduct pairwise significance tests of prediction errors among the models (43). When performing the Diebold–Mariano (DM) test, the same rolling time window mechanism was used for empirical testing.

A rolling time window validation was adopted to evaluate the predictive performance of the models. The full dataset comprised 168 months of monthly HIV incidence data from January 2012 to December 2025. The training window was set to 60 months, the forecasting window to 12 months, and the rolling step size to 12 months. The initial training set covered January 2012 to December 2016, forecasting January to December 2017. Subsequently, both the training and forecasting windows were rolled forward synchronously by 12 months each time, resulting in a total of 9 rolling iterations covering the period 2017–2025. At each iteration, the model was trained using only historical data without incorporating any future information.

In our study, we split the dataset chronologically into a training set (first 60 months, Jan 2012–Dec 2016), a validation set (middle 36 months, Jan 2017–Dec 2019), and an independent test set (Jan 2020–Dec 2025) to ensure internal validation. We performed all hyperparameter tuning on the validation set, and we reported rolling-window results on the test set to enable prospective evaluation.

All models were trained and evaluated under the same rolling window settings to ensure fair comparison. Predictive performance was quantified using the following metrics: root mean squared error (RMSE), mean absolute error (MAE), mean absolute percentage error (MAPE), symmetric mean absolute percentage error (sMAPE), and mean absolute scaled error (MASE). Lower values of these indicators correspond to higher prediction accuracy. The calculation formulas are as follows (19):

$$\text{MAE}-\frac{\sum_{t=1}^{n}\left|X_{t}-{\hat{X}}_{t}\right|}{n}$$

$$\text{RMSE}=\sqrt{\frac{\sum_{t-1}^{n}{\left(X_{t}-{\hat{X}}_{t}\right)}^{2}}{n}}$$

$$\text{MAPE}=\frac{1}{n}\sum_{t=1}^{n}\left|\frac{X_{t}-{\hat{X}}_{t}}{X_{t}}\right|$$

$$\text{sMAPE}=\frac{1}{n}\sum_{t=1}^{n}\frac{2\left|X_{t}-{\hat{X}}_{t}\right|}{\left|X_{t}\right|+\left|{\hat{X}}_{t}\right|}\times 100\%$$

$$\text{MASE}=\frac{\frac{1}{n}\sum_{t=1}^{n}\left|X_{t}-{\hat{X}}_{t}\right|}{\frac{1}{n-1}\sum_{t=2}^{n}\left|X_{t}-{\hat{X}}_{t-1}\right|}$$

Where ${\hat{X}}_{t}$ is the predicted value, *X*_*t*_ is the observed value at time t and n is the sequence sample size. For MASE, the denominator is the mean absolute error of the naive forecast.

To quantify prediction uncertainty, Bootstrap resampling (1,000 iterations) combined with rolling window validation was applied to calculate the 95% confidence intervals for RMSE, MAE, MAPE, sMAPE and MASE for each model.

### Data analysis

The R software version 4.2.2 was applied to construct the SARIMAX, Prophet, and random forest (RF) models. The forecast package provided the arima() function for SARIMAX (with exogenous variables). The prophet package builds prophet models with exogenous variables, and the random forest package builds random forest models. The level of significance was set at *p* < 0.05.

## Results

### Time series characteristics of monthly HIV incidence in Xinjiang

In this study, the results showed that the monthly HIV incidence rate in Xinjiang Uygur Autonomous Region ranged from 0.0348 to 1.8020 per 100,000 population during the period from January 2012 to December 2025. The median was 0.5862, the variance was 0.0791, the interquartile range was 0.3395, the skewness was 0.6716, and the kurtosis was 1.2885. Over the entire study period, the overall HIV incidence exhibited a decreasing trend characterized by an initial increase, a subsequent stabilization, and a final fluctuation (Figure 1). The monthly incidence data also revealed distinct periodicity and seasonality. Seasonally, the trough period (January–February) represented the lowest values of the year; March saw a rapid rise to the highest peak of the seasonal cycle (Figure 2).

**Figure 1:**
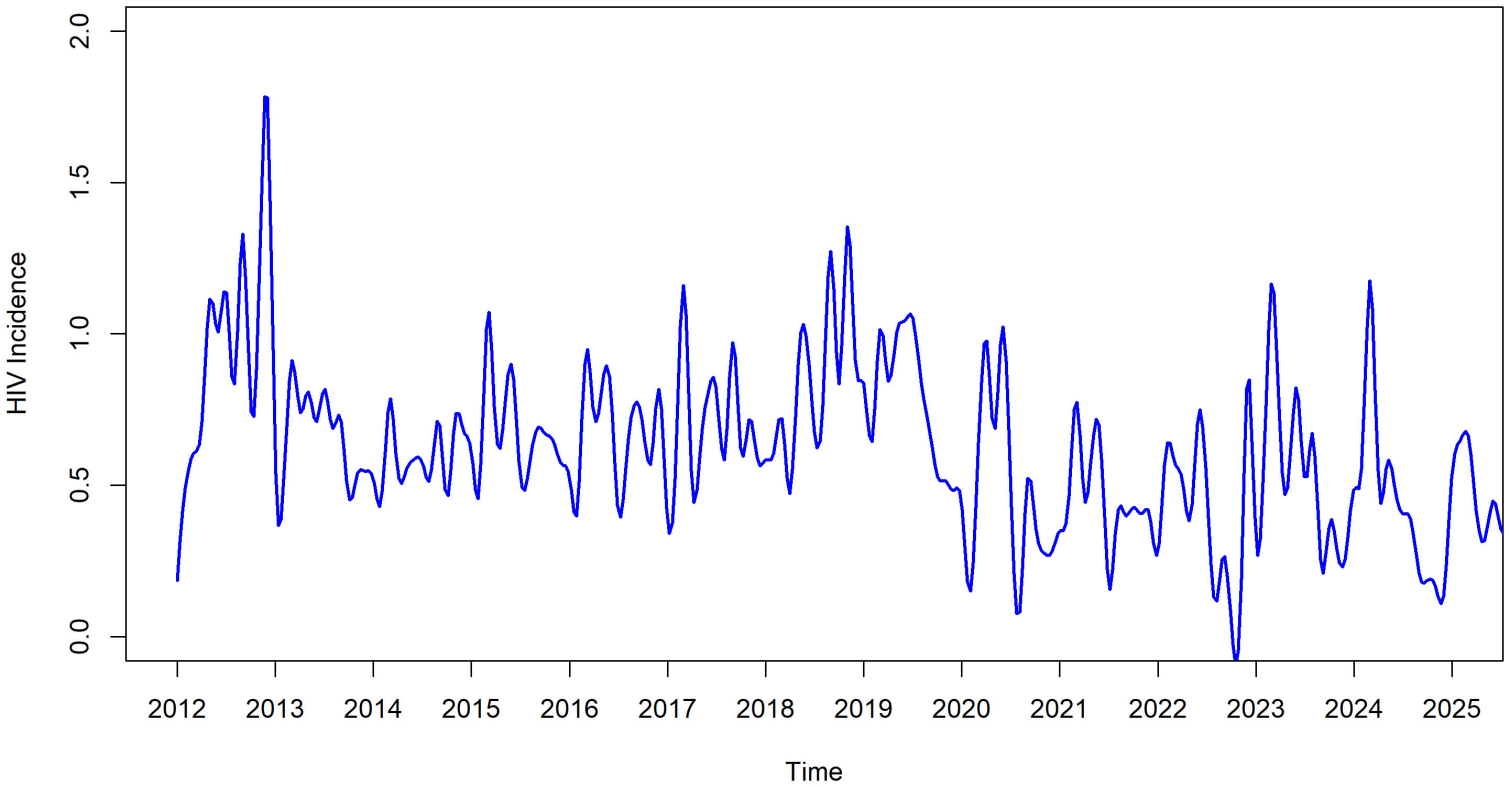
Original time series plot of HIV incidence in Xinjiang, 2012–2025.

**Figure 2:**
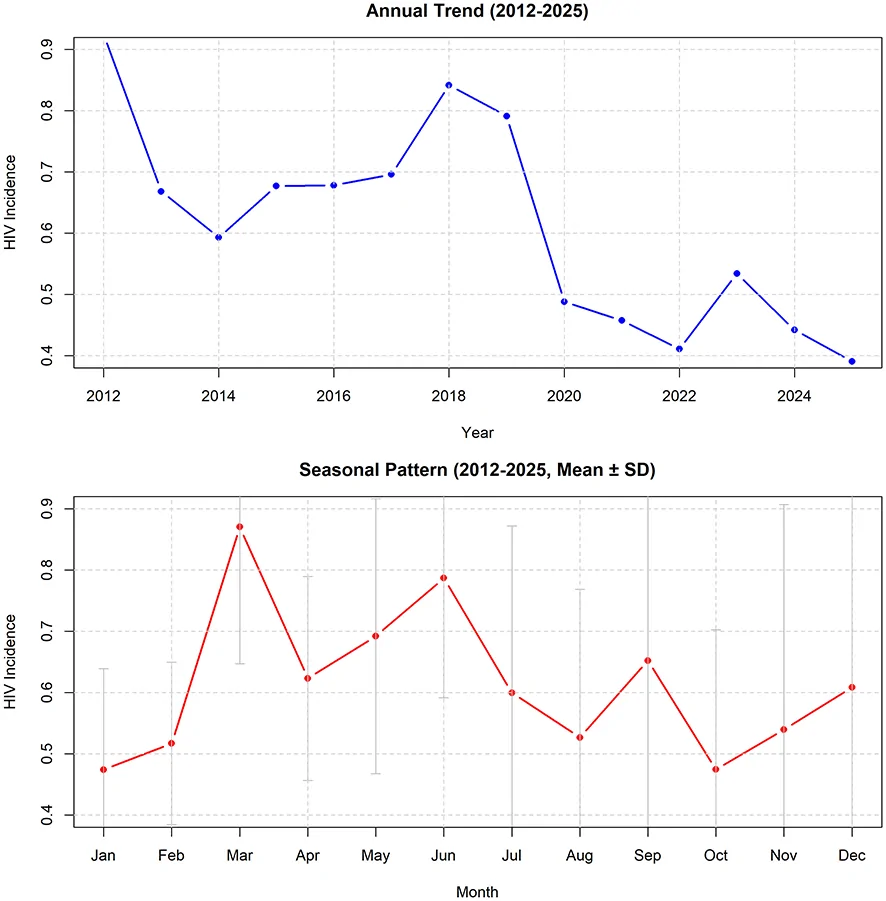
Cyclical changes and seasonal distribution characteristics of HIV incidence in Xinjiang Uygur Autonomous region, 2012–2025.

To assess the robustness of this seasonal pattern, we performed STL decomposition and compared monthly mean incidence with and without the COVID-19 years (2020–2022); (Figure 3). The March peak and January-February trough persisted after excluding the pandemic years, confirming that the observed seasonality was robust (Figure 3).

**Figure 3:**
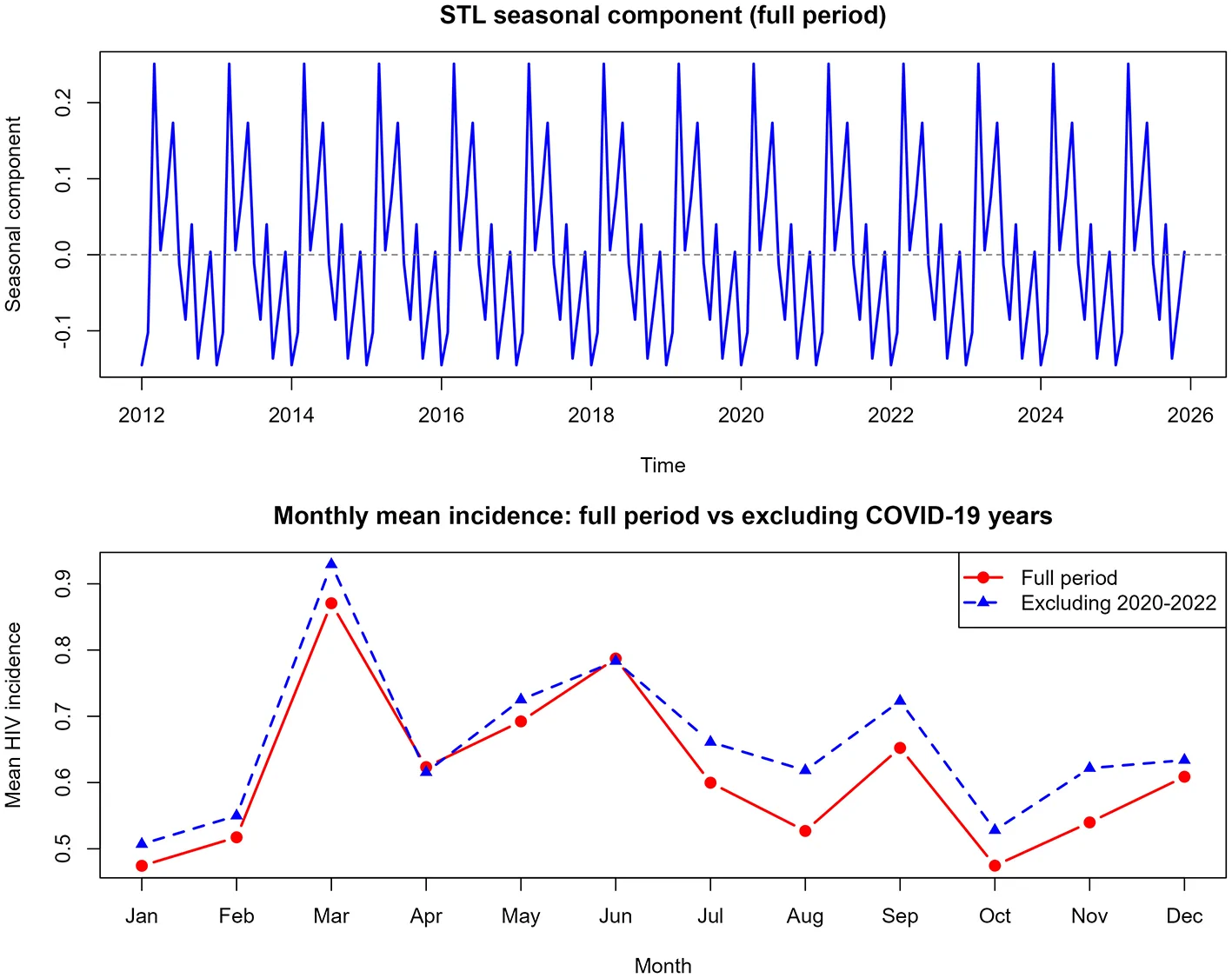
Seasonality robustness check of monthly HIV incidence in Xinjiang.

### SARIMAX model

In this study, the auto.arima() function from R version 4.2.2 was employed to perform automated modeling and selection on the monthly HIV incidence data from January 2012 to December 2025. First, after first-order differencing the ADF test confirmed that the series became stationary (Dickey–Fuller statistic = −7.6398, *p* = 0.01). Second, the auto.arima() function automated the model search and selection, incorporating an exogenous variable (COVID-19 intervention) and yielding the optimal model as SARIMA(0,1,4)(1,0,0)(1) + exogenous. Third, the Box–Jenkins *Q*-test applied to the residuals produced a Q statistic of 8.81 (*p* = 0.7199), indicating that the residuals constituted white noise. Finally, the selected model achieved an AIC of −21.04 and a BIC of 0.79. The COVID-19 intervention coefficient was −0.1615 (SE = 0.0857, *p* = 0.0596), with a 95% confidence interval of [−0.3295, 0.0065]. Given the white noise residuals and these information criteria, the model was considered adequate. Detailed model parameters and diagnostics are shown in Table 1 and Figure 4.

**Table 1:** Model parameters and white noise test for SARIMA(0,1,4)(1,0,0)(1) + exogenous.

Parameter	Coefficient
MA(1) coefficient	−0.7083
MA(2) coefficient	−0.0826
MA(3) coefficient	0.1098
MA(4) coefficient	−0.2452
SAR(1) coefficient	0.3859
COVID-19 intervention coefficient	−0.1615

**Figure 4:**
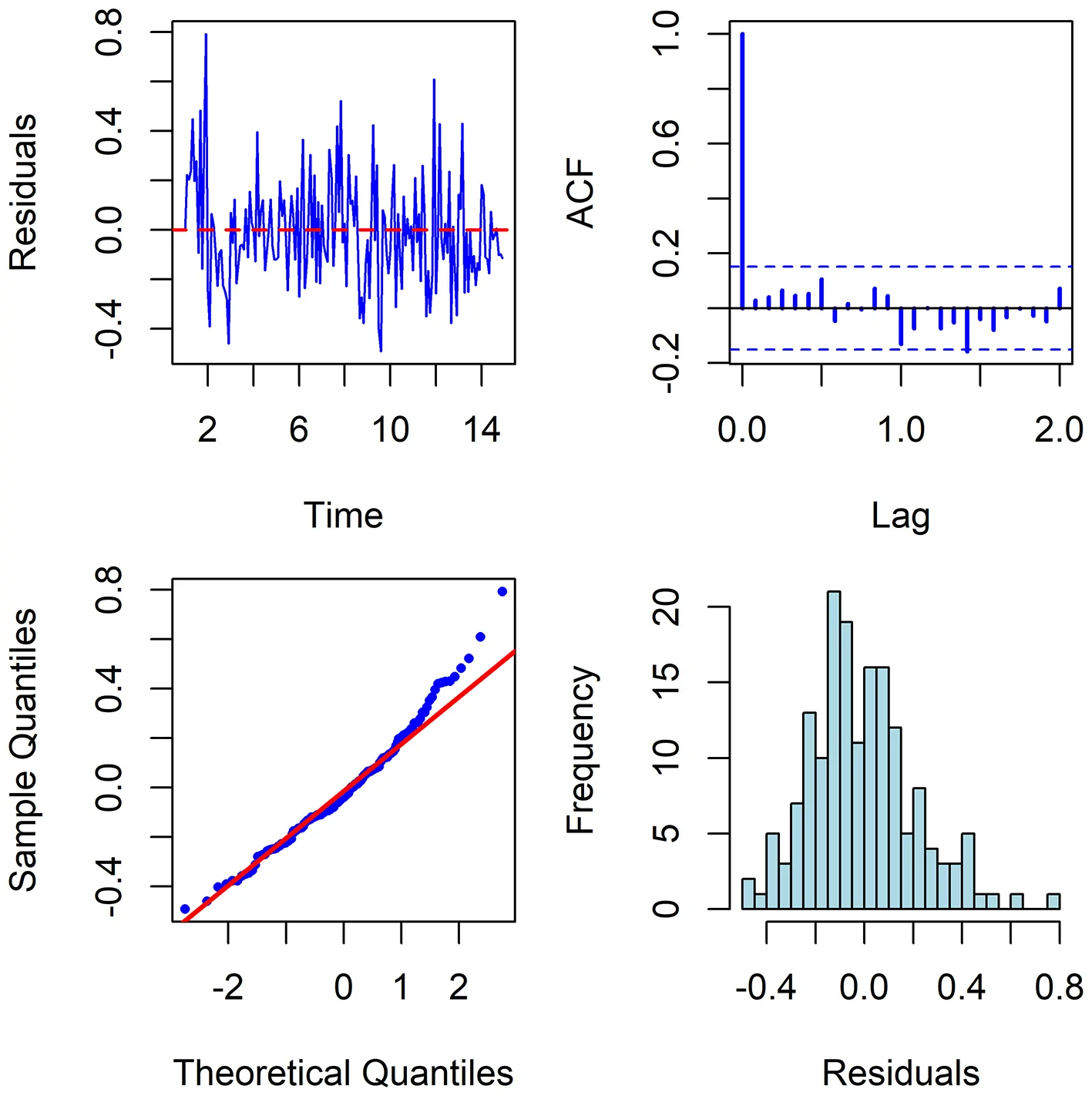
Residual diagnostics (ACF and normal Q-Q plot) for the SARIMA(0,1,4)(1,0,0)(1) + exogenous model.

### Prophet model with exogenous variables

In this study, the “prophet” package was implemented in R to fit a prophet model with exogenous variables. Given that HIV incidence was weakly associated with holiday effects, the model did not explore the predictive influence of holidays on incidence. The optimal model was an additive regression model with piecewise linear trend and 25 change points, incorporating COVID-19 as an exogenous variable. The estimated marginal effect of the COVID-19 intervention was −0.0320 (MAP estimate). Since the default prophet model does not provide standard errors or *p*-values, we further performed Bayesian MCMC inference (1,000 posterior samples). The posterior mean was −0.1064 (SE = 0.0281), with a 95% confidence interval of [−0.1588, 0.0495], and a two-sided posterior probability *p* = 0.001, indicating that the COVID-19 intervention had a statistically significant negative effect on HIV incidence. Predictions were generated as shown in Figure 5. The shaded area in Figure 5 represents the 95% prediction interval based on the MCMC posterior distribution.

**Figure 5:**
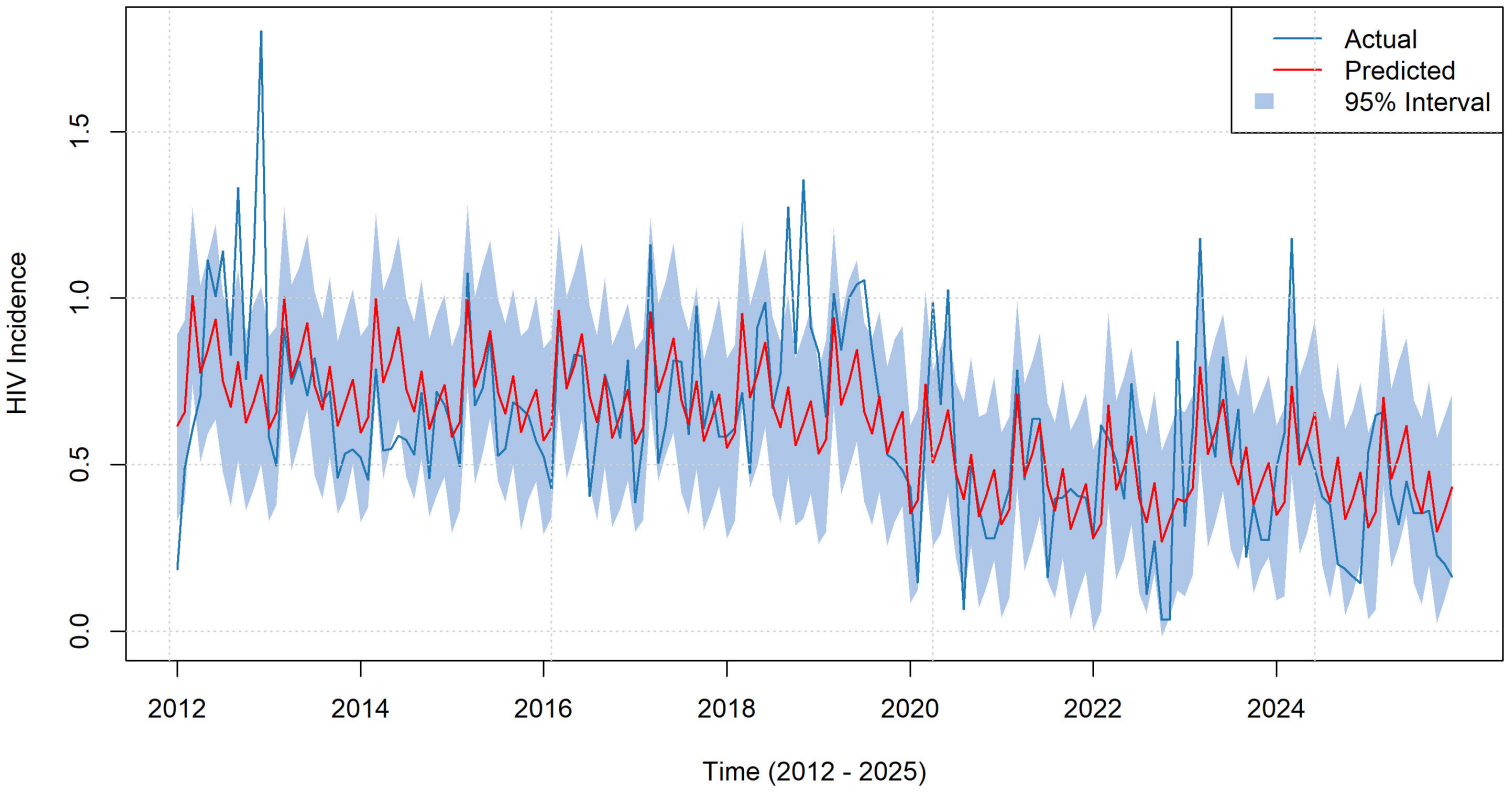
Comparison of actual and predicted HIV incidence in Xinjiang. Solid blue line: actual incidence; red line: predicted values; shaded area: 95% prediction interval based on MCMC posterior distribution.

### Random forest

We used the monthly HIV incidence rate as the dependent variable and selected its 12 lagged values along with the COVID-19 intervention variable and month as features to build a random forest regression model. A random seed(123) was set, and 500 regression trees were grown, with the number of variables tried at each split (mtry) and the minimum node size both set to their default values. The mtry parameter was tuned using the tuneRF() function, and the optimal value was 3. Model fitting results showed that the random forest achieved a root mean square error (RMSE) of 0.0866, a mean absolute error (MAE) of 0.0652, and a mean absolute percentage error (MAPE) of 20.89%. In the model, the COVID-19 intervention variable had a permutation importance (%IncMSE) of 4.31%, ranking 10th among 14 features, indicating a limited independent contribution to predictive accuracy and that model performance mainly relied on historical lags of HIV incidence. SHAP analysis showed that COVID-19 ranked 14th (last) with a mean absolute SHAP value of 0.00135, substantially lower than the top-ranked features: lag_1 (0.00869), lag_8 (0.00627), and lag_3 (0.00563); (Table 2).

**Table 2:** SHAP feature importance ranking for the Random Forest model.

Rank	Feature	SHAP importance
1	lag_1	0.00869
2	lag_8	0.00627
3	lag_3	0.00563
4	lag_5	0.00521
5	month	0.00397
6	lag_4	0.00297
7	lag_10	0.00293
8	lag_9	0.00263
9	lag_11	0.00247
10	lag_7	0.00170
11	lag_2	0.00166
12	lag_6	0.00156
13	lag_12	0.00156
14	Covid-19	0.00135

Features are ranked by mean absolute SHAP values; higher values indicate greater contribution to the model's predictions. SHAP importance scores are calculated as the mean absolute SHAP values across all instances.

Figure 6 shows a SHAP beeswarm plot of the random forest model. Each point represents one sampled observation; the x-axis indicates the SHAP value, the y-axis indicates the feature, and the color represents the feature value. Points farther to the right indicate a greater positive contribution of the feature to the prediction, while points farther to the left indicate a greater negative contribution.

**Figure 6:**
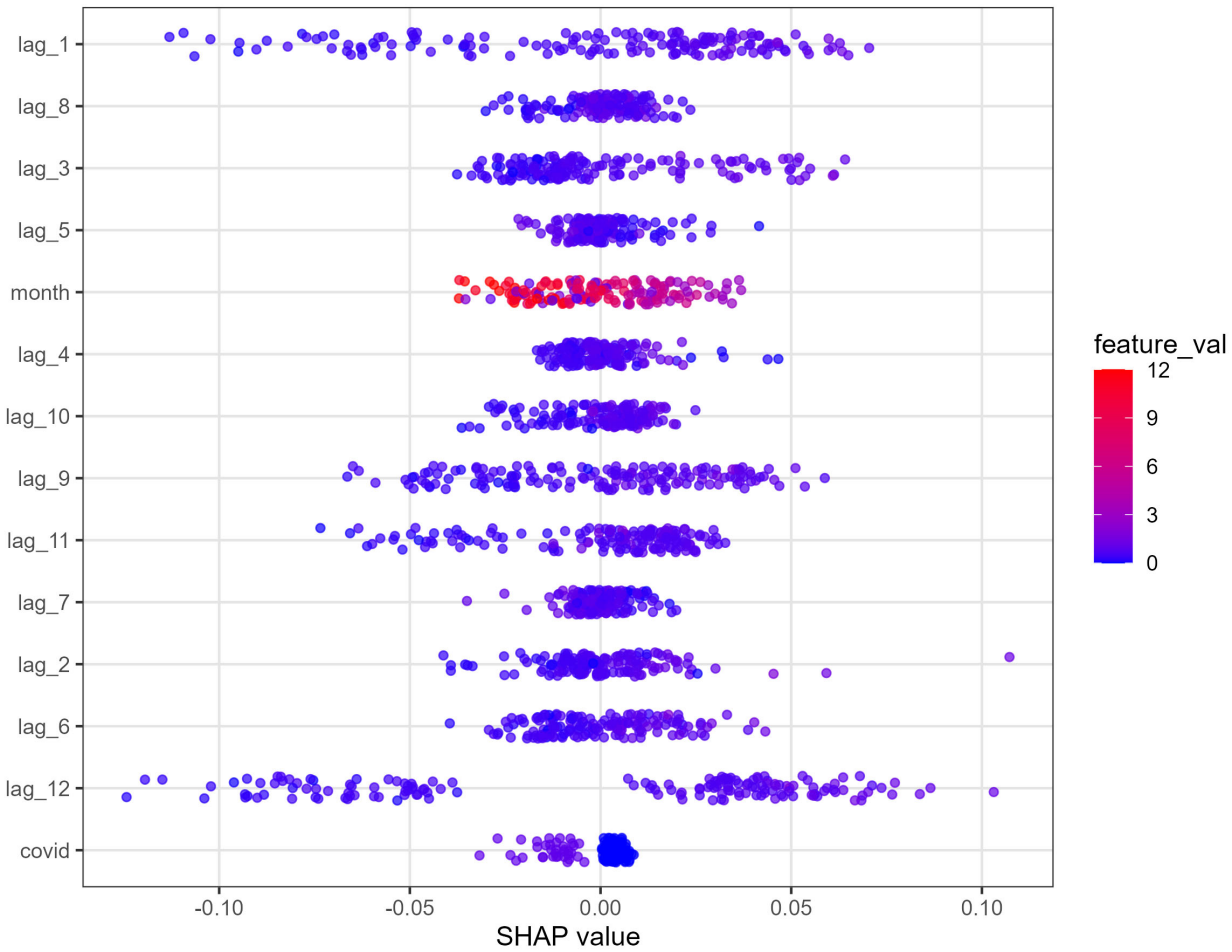
SHAP beeswarm plot of feature contributions in the Random Forest model for monthly HIV incidence in Xinjiang.

### Prediction performance evaluation

In rolling validation, SARIMAX failed to produce valid forecasts for windows 1–4 (forecast years 2017–2020). When the fixed SARIMAX(0, 1, 4)(1, 0, 0) (1) model with the exogenous variable was refitted, the optimizer returned infinite values during maximum likelihood estimation, and the model failed to converge. The specific R error was “Error in optim(init[mask], armaCSS, method = optim.method, hessian = FALSE,: optim returned infinite values”, and the corresponding loop output was “Window 1 refit failed: optim returned infinite values”, followed by “Window 1 skipped”; the same error and skip occurred for windows 2, 3, and 4. For these failed windows, no model object was generated; consequently, no parameter estimates, standard errors, or forecast values were available, and no replacement or imputation was performed. Windows 5–9 (forecast years 2021–2025) converged successfully, yielding 5 successful windows and 60 forecast values, with an RMSE of 0.2436, an MAE of 0.1899, and a MAPE of 96.68%. Therefore, the three models were compared only on windows 5–9. These windows still constituted a prospective evaluation because each model was trained exclusively on data prior to the forecast year, without incorporating future information. However, the exclusion of windows 1–4 may introduce selection bias, and the model comparison should be interpreted as restricted to the successfully converged windows.

To ensure a fair comparison, all three models were evaluated on windows 5–9 (corresponding to 2021–2025). The results showed that RF achieved the lowest RMSE (0.2211), MAE (0.1722), and MAPE (85.83%), compared to SARIMAX (0.2436, 0.1899, 96.68%) and Prophet with exogenous variables (0.2258, 0.1779, 88.70%); (Table 3). However, these numerical differences were not statistically significant (Table 4).

**Table 3:** Comparison of predictive performance of different models in rolling validation (windows 5–9).

Models	RMSE	MAE	MAPE	sMAPE	MASE
SARIMAX	0.2436	0.1899	96.68%	43.24%	0.9751
95% CI	[0.1956, 0.2831]	[0.1504, 0.2279]	[51.17%, 164.60%]	[33.58%, 53.02%]	[0.7820, 1.1685]
Prophet model with exogenous variables	0.2258	0.1779	88.70%	43.77%	0.9486
95% CI	[0.1834, 0.2671]	[0.1421, 0.2169]	[43.39%, 151.25%]	[34.59%, 53.30%]	[0.7738, 1.1380]
Random Forest	0.2211	0.1722	85.83%	41.11%	0.8745
95% CI	[0.1735, 0.2720]	[0.1387, 0.2102]	[41.86%, 142.37%]	[32.12%, 50.70%]	[0.7138, 1.0779]

95% confidence intervals were estimated via Bootstrap resampling with 1,000 iterations based on rolling window forecast errors (60 observations for windows 5–9). MAPE is sensitive to near-zero monthly incidence values, because small absolute errors in these months produce disproportionately large percentage errors and may overstate forecast error; therefore, RMSE, MAE, sMAPE, and MASE were considered the primary metrics. sMAPE and MASE were consistent with RMSE and MAE, confirming that the high MAPE was driven by near-zero observations rather than poor model performance.

**Table 4:** Diebold–Mariano test results of different models (windows 5–9).

Models comparison	DM statistic	*p*-value	95% CI for DM	RMSE difference
SARIMAX vs. Prophet model with exogenous variables	0.5173	0.6069	[−1.9404, 4.0909]	0.0178
SARIMAX vs. random forest	1.1423	0.2579	[−0.8930, 5.8604]	0.0225
Prophet model with exogenous variables vs. random forest	1.4330	0.1571	[−1.2806, 5.1637]	0.0047

Bonferroni corrected significance level: α = 0.05/3 = 0.0167. All p-values are greater than 0.0167, indicating no statistically significant difference in predictive accuracy between any two models. The DM test was based on 60 forecast errors (windows 5–9, df = 59); the limited number of forecast errors reduces statistical power to detect moderate differences.

The relatively high MAPE for Random Forest (85.83%) was primarily attributable to near-zero monthly incidence values, which disproportionately inflated percentage errors. To further evaluate this, sMAPE and MASE were calculated for Random Forest, yielding 41.11% and 0.8745, respectively. Both were consistent with its low RMSE and MAE, confirming that the high MAPE was driven by near-zero observations rather than poor model performance.

Forecast calibration was not performed due to the limited out-of-sample forecast horizon (60 months), which provides insufficient data points for robust calibration assessment.

To compare the predictive accuracy of the three models, we employed the Diebold–Mariano test. The results show that after pairwise comparisons with Bonferroni correction for multiple testing, there is no statistically significant difference in predictive accuracy between any two models (Table 4).

## Discussion

This study systematically evaluated the prospective performance of three models for HIV incidence forecasting in Xinjiang using a rolling validation framework. The main findings of this study are summarized in three aspects. First, monthly HIV incidence in Xinjiang showed distinct periodicity and seasonality, with a trough in January-February and a peak in March. Overall incidence exhibited a decreasing trend characterized by an initial increase, subsequent stabilization, and final fluctuation. Second, the three models showed divergent estimates of the COVID-19 effect: SARIMAX and Prophet detected negative effects with varying levels of significance, while the COVID-19 variable made only a limited contribution in the random forest model. Third, rolling validation showed that random forest achieved the lowest numerical error metrics within the successfully converged windows; however, the Diebold–Mariano test with Bonferroni correction found no statistically significant differences among the three models, and the numerical differences were small. Thus, the three models were statistically indistinguishable in this study.

To interpret these results, we consider the drivers of seasonality and the principles of the three models. Regarding seasonality, the study found that HIV incidence in Xinjiang exhibited distinct seasonal and periodic patterns. Unlike respiratory infectious diseases, these patterns were not caused by inherent seasonal dependence of the HIV virus itself. Instead, they resulted from the combined effects of multiple factors. The seasonal and periodic patterns of HIV incidence in Xinjiang may be associated with the following factors. First, population behavioral patterns and climatic factors may play a role. Research indicates that population behavioral patterns influence infectious disease transmission (44). Xinjiang has a vast and sparsely populated territory with high population mobility that correlates significantly with seasonal changes. For example, seasonal employment leads to alternating dispersion and aggregation of the population, and such seasonal clustering may potentially affect HIV transmission (45). In addition, population mobility and cross border factors in Xinjiang represent another important consideration. Existing studies have identified a certain epidemic trend of HIV infection among individuals entering or exiting Xinjiang at ports of entry, where passenger traffic volume fluctuates seasonally due to peak tourist seasons and festive events (46). Second, seasonal testing behavior is a major contributor to the seasonal variation in newly diagnosed HIV cases. Existing studies have demonstrated that the seasonal pattern observed in reported HIV cases primarily results from seasonal fluctuations in testing behavior (47). Third, the spatiotemporal distribution of HIV epidemics in Xinjiang influences the periodic patterns of local reported AIDS cases (48). For example, spatial analysis has predicted Xinjiang as a new hotspot for HIV incidence, primarily attributed to a spatiotemporal coupling between seasonal population mobility and interregional transmission risk (49, 50). Through this coupling, periodic fluctuations from external high incidence regions are imported via migration routes and combined with intensive local testing interventions, which significantly amplifies both the seasonal peak and the periodic amplitude of reported HIV cases in Xinjiang.

Given the strong seasonality and periodicity of the HIV incidence data, SARIMAX, Prophet (19, 38), and random forest are all well suited for capturing these temporal features (41, 42), making them appropriate choices for the research question. Nevertheless, their performances diverged in two key aspects: the estimation of the COVID-19 effect and the numerical accuracy in rolling validation.

First, regarding the COVID-19 effect, we also found that the COVID-19 effect was significant or weakly significant in SARIMAX and Prophet but was not important in the random forest model. This result may be explained by the different mechanisms through which the three models process information. For example, SARIMAX and Prophet can directly incorporate an exogenous variable (the COVID-19 variable) and directly estimate its effect (34). In contrast, random forest treats the exogenous variable (the COVID-19 variable) as a feature (42). Consequently, the contribution of COVID-19 may be diluted by historical lagged values, and its independent contribution may be distributed among other features, thereby weakening the effect of the exogenous variable.

This COVID-19 binary variable exhibited consistently low importance in both permutation importance and SHAP analyses, which reflects the distinctive epidemiological characteristics of HIV. Unlike acute respiratory infections such as influenza, HIV is a chronic infection with a prolonged incubation period. Short-term disruptions in healthcare accessibility or testing behavior may primarily affect case detection rather than true incidence, and such effects may be attenuated when aggregated at the monthly level. Moreover, the limited contribution of the binary COVID-19 indicator may also reflect the constraint of using a crude binary variable; other potential covariates, including HIV testing volume (47), mobility indices, and stringency measures, could have better captured pandemic-related disruptions but were not included in this study due to data unavailability.

The study also found that although both SARIMAX and Prophet yielded negative point estimates for the COVID-19 effect, their significance levels differed. This discrepancy may be attributable to differences in model structure, prior specification, and trend flexibility (33, 51). SARIMAX estimates the exogenous effect conditional on an ARMA error structure and a seasonal term (40), which may absorb part of the intervention effect and inflate the standard error, resulting in marginal significance. In contrast, Prophet uses a Bayesian framework with a flexible piecewise linear trend (51) and 25 change points, allowing the trend to capture gradual changes while the exogenous regressor estimates the remaining effect. The narrower posterior standard error in Prophet may partly reflect the influence of the default prior and the MCMC sampling procedure. In addition, the binary coding of the COVID-19 variable (2020–2022 = 1) is a crude approximation and may be differentially absorbed by the trend and seasonal components across models. Therefore, the divergent significance levels should be interpreted as model dependent rather than as evidence of a consistent causal effect.

Second, because the three models were statistically indistinguishable in rolling validation, model selection should be guided by interpretability, data-updating frequency, and resource constraints rather than by marginal accuracy differences. This observation may be attributed to the differing underlying principles of the three modeling approaches. SARIMAX and Prophet are capable of capturing the core temporal features of HIV incidence, including trend, periodicity, and seasonality (34, 38), however, they rely on relatively strong linear or decomposable structural assumptions (51–54), which limit their ability to capture nonlinear patterns in the HIV incidence data. In contrast, random forest depends more on historical lagged values of the series for prediction and is well suited for modeling the complex nonlinear relationships inherent in the HIV incidence time series (41, 42).

The relatively lower numerical errors of random forest within the successfully converged windows can be further attributed to several factors (1). As a tree-based ensemble method, Random Forest naturally captures nonlinear interactions and higher-order dependencies among lagged incidence values without requiring a prespecified functional form, rendering it well suited to HIV incidence data, which frequently exhibit reporting delays, sporadic outbreak clusters, and complex seasonal patterns (2). Random Forest is robust to outliers and does not assume linearity or stationarity (41, 42), affording greater flexibility than SARIMAX and Prophet in predicting HIV incidence (3). Our SHAP analysis identified short-term lagged terms (lag_1, lag_3, and lag_8) as the most influential predictors, suggesting that HIV transmission dynamics are primarily driven by auto-regressive momentum. This finding is consistent with the epidemiological understanding that HIV incidence is largely sustained by ongoing transmission networks and testing behaviors, both of which possess inherent temporal persistence.

Notably, lag_8 ranked second in SHAP importance, which may reflect an approximately 8 month cycle in HIV reporting, testing, or follow-up. Because HIV is a chronic infection with a long and variable incubation period, newly reported cases often reflect infections acquired months earlier. This reporting characteristic may further amplify the influence of seasonal mobility and periodic testing activities on reported incidence. However, this interpretation remains exploratory because HIV testing volume and mobility data were not included in the model. Future studies with longer time series and additional covariates should examine whether this 8 month periodicity is robust.

Furthermore, the Diebold–Mariano test with Bonferroni correction confirmed that no model significantly outperformed the others. This may be because the numerical advantage of Random forest was limited and the small number of rolling windows reduced statistical power. These results reinforce that the three models performed comparably in this study.

Based on the above main findings and the analysis of underlying reasons, this study offers the following policy implications. First, epidemiological implications: our findings indicate that HIV incidence in Xinjiang follows a distinct seasonal pattern, with a peak in March and a trough in January-February. This period corresponds to late winter and early spring, a time of alternating holidays and increased population mobility. The robustness of this seasonal pattern was confirmed by STL decomposition and after excluding the COVID-19 years. Therefore, strengthening HIV education and implementing seasonal HIV testing are effective prevention and control strategies (47). Specifically, HIV prevention and education should be intensified (55). Awareness campaigns should be conducted in schools, communities, entertainment establishments, shopping malls, and other public settings to help the public understand HIV prevention and response knowledge. In addition, health authorities should plan resource allocation in advance. For seasonal HIV testing, human and material resources (e.g., testing supplies, personnel) should be properly allocated, and coordination should be established with hospitals and CDCs to ensure integrated implementation (56).

Second, public health application recommendations: strengthen surveillance and early warning of HIV incidence. Incorporate the recognition of seasonal patterns into the surveillance and early warning system to enable proactive warning and early intervention before the epidemic peak, thereby providing a time window for precision prevention and control. SARIMAX, Prophet, and Random forest can all be considered as tools for HIV incidence surveillance and early warning; however, data should be updated continuously and models should be revised regularly. Model selection should be based on the trade-off between data characteristics, interpretability, data-updating frequency, and resource constraints to select the most appropriate model for predicting and assessing HIV incidence trends (57).

Our study has several limitations. First, traditional time series models such as ETS were excluded from our analysis because they cannot incorporate exogenous variables such as the COVID-19 intervention. Although this decision followed the model's inherent limitation, it may affect the comprehensiveness of model comparison in real-world complex settings. Second, HIV incidence is influenced by multiple interacting factors including social, economic, and population characteristics (58). However, these factors were not incorporated into the forecasting models, which limits the interpretability of the predictions and prevents a full explanation of the logical relationships between various determinants and HIV incidence. Third, formal structural break analysis using CUSUM or Bai–Perron was not performed due to the limited sample size (168 months). Fourth, since the SARIMAX model already incorporates the exogenous COVID-19 intervention variable, we did not adopt additional hybrid models, to avoid unnecessary complexity and the risk of overfitting given our sample size. Fifth, SARIMAX failed to converge in the first four rolling windows (2017–2020), so model comparison was restricted to windows 5–9 (2021–2025), which may introduce selection bias. Sixth, HIV reporting delay and data revision may affect monthly counts and forecast accuracy, and these effects could not be fully avoided. Seventh, newly reported HIV cases were used as a proxy for HIV incidence; however, newly reported cases are not equivalent to new infections, because some cases may have been infected earlier and diagnosed later. Eighth, the model retraining frequency was fixed at 12 months in the rolling window design, and different retraining frequencies may affect forecast performance. Finally, as data on relevant covariates such as mobility and testing behavior could not be obtained for this study, the interpretations of these factors in the discussion lack direct empirical support.

Therefore, future studies should incorporate relevant influencing factors into prediction models, including socioeconomic conditions, population behavior (e.g., mobility and testing behavior), patient healthcare seeking behavior, and healthcare resource allocation, HIV testing volume, mobility indices, and ART coverage. HIV testing volume should be used to better distinguish new infections from newly diagnosed cases, and different model retraining frequencies should be evaluated to assess their impact on forecast accuracy. Future studies should also explore alternative estimation methods or sensitivity analyses for SARIMAX so that the full evaluation period can be assessed. Reporting delay and data revision should also be quantified and adjusted for in future forecasting studies. Additionally, structural break detection methods such as CUSUM or Bai–Perron should be applied when longer time series become available, to systematically assess breakpoints and their impact on forecast accuracy. Data should be continuously updated in real time, and models should be constructed using continuous time series data. Such an approach will further enhance prediction accuracy and scientific rigor, thereby providing more reliable technical support for precise HIV epidemic control.

## Conclusion

This study demonstrates that monthly HIV incidence data in Xinjiang exhibit significant seasonality and periodicity. The SARIMAX, Prophet with exogenous variables, and random forest models all provide reasonable predictions of HIV incidence while capturing these temporal features. After hyperparameter tuning via grid search, random forest achieved the lowest numerical error metrics within the successfully converged rolling windows; however, the Diebold–Mariano test with Bonferroni correction found no statistically significant differences among the three models. Because SARIMAX failed to converge in four windows and the numerical differences were small, the three models should be considered statistically indistinguishable in this study. SHAP analysis further revealed that short-term lagged incidence values were the dominant predictors, while the COVID-19 binary variable contributed minimally, reflecting the chronic nature of HIV transmission. Therefore, model selection for HIV incidence prediction in Xinjiang should be guided by the specific application context, balancing interpretability, data-updating frequency, and resource constraints rather than marginal accuracy differences.

## Data Availability

The original contributions presented in the study are included in the article/supplementary material, further inquiries can be directed to the corresponding author.
